# tidybulk: an R tidy framework for modular transcriptomic data analysis

**DOI:** 10.1186/s13059-020-02233-7

**Published:** 2021-01-22

**Authors:** Stefano Mangiola, Ramyar Molania, Ruining Dong, Maria A. Doyle, Anthony T. Papenfuss

**Affiliations:** 1 Bioinformatics Division, Walter and Eliza Hall Institute of Medical Research, Parkville, VIC Australia; 2 Department of Medical Biology, University of Melbourne, Melbourne, VIC Australia; 3 Peter MacCallum Cancer Centre, Melbourne, VIC 3000 Australia; 4 Sir Peter MacCallum Department of Oncology, University of Melbourne, Melbourne, VIC Australia; 5 School of Mathematics and Statistics, University of Melbourne, Melbourne, VIC 3010 Australia

## Abstract

Recently, efforts have been made toward the harmonization of transcriptomic data structures and workflows using the concept of data tidiness, to facilitate modularisation. We present tidybulk, a modular framework for bulk transcriptional analyses that introduces a tidy transcriptomic data structure paradigm and analysis grammar. Tidybulk covers a wide variety of analysis procedures and integrates a large ecosystem of publicly available analysis algorithms under a common framework. Tidybulk decreases coding burden, facilitates reproducibility, increases efficiency for expert users, lowers the learning curve for inexperienced users, and bridges transcriptional data analysis with the tidyverse. Tidybulk is available at R/Bioconductor bioconductor.org/packages/tidybulk.

**Supplementary Information:**

The online version contains supplementary material available at 10.1186/s13059-020-02233-7.

## Background

High-throughput decoding of RNA genetic material has proved to be a disruptive tool for the understanding of dynamic biological systems. Bulk and single-cell RNA sequencing provides a large amount of information about gene transcript abundance, transcriptional rate, and genetic heterogeneity, as well as allelic information and gene fusions. During the last decades, the scientific community has built a rich ecosystem of algorithms for the exploration and the analysis of transcriptomics data. The R programming language [1] has been a vital part of this ecosystem, with the repositories CRAN and Bioconductor [2] hosting many of these algorithms. Despite the use of central repositories, algorithms included in common analyses workflows are based on a diverse range of input and output data structures including sample-oriented and transcript-oriented data frames, matrices and custom S3 and S4 objects. This poses unique challenges for data transformation and data integration along workflows, which are two error-prone processes. Some efforts have been made to build a robust and standardized data structure that holds heterogeneous information. For example, SummarizedExperiment [3] integrates a matrix-like object of transcript abundance, a sample- and a transcript-oriented metadata data frames.

More recently, the R data analysis community has made a collective endeavor toward the harmonization of data structures and workflows using the concept of tidiness [4]. The goals of tidy data frames are the ease of manipulation, modeling, and visualization and are characterized by having a specific structure where each variable is a column, and each observation is a row. This paradigm is extremely powerful when analyzing and manipulating biological data, because it directly captures how biological data measurements relate to experimental design and metadata (e.g., technical and clinical properties of transcripts and replicates). The adoption of a tidy and consistent data frame makes it easier for the community to develop modular manipulation, visualization, and analysis tools that are endomorphic (i.e., they return the same type as their input). For example, the dplyr and tidyr packages [5, 6] map notions of information manipulation to verbs that act on tidy data. These verbs can be assembled into a workflow using a pipe operator (%>%) [7] that effectively streams a data frame through all processes. This integrated and modular framework enables robust, reproducible, error-resistant, human-readable workflows that can benefit the scientific community. The concept of tidiness has already been applied to other areas of data analysis in genomics. The package plyranges [8] introduced a dplyr-like interface for interacting with some of the most common data structures containing genomic coordinates including Ranges and GenomicRanges [9]. For example, it allows the filtering of genes in genomic intervals. The package organism.dplyr introduced a dplyr-like interface for annotation packages [10]. It supports the creation of gene annotation databases for a wide range of organisms. The ggplot grammar of graphics was extended to genomics data by ggbio [11], allowing for example the production of annotated chromosome tracks. While the current ecosystem covers several aspects of genetic data manipulation, analysis, and visualization, transcript abundance analysis remains uncovered. Here we present tidybulk, a modular framework for bulk transcriptional analyses based on a tidy data structure paradigm and a user-friendly grammar that underlies a large selection of publicly available tools for transcriptional analyses [12–29]. The main aim of this study is to bridge the ecosystem of transcriptional data analysis with the tidy ecosystem (i.e., tidyverse). The procedures covered by tidybulk include the quantification of transcript abundance from genome mapping, identification of abundant and variable transcripts, data scaling and adjustment, duplicates aggregation, dimensionality reduction, sample-wise and gene-wise redundancy elimination, clustering, differential gene transcriptional abundance and gene enrichment testing, cellularity deconvolution, and differential tissue composition testing.

Tidybulk is highly complementary to the existing ecosystem. While plyranges improves manipulation of genomic coordinate data frames (which are tidy in nature), tidybulk tackles transcriptomic data representation introducing a novel and tidy structure and provides a framework that spans all stages of analysis using unified grammar. This framework allows quick-to-produce and flexible workflows. Specific goals of tidybulk are (i) to decrease the coding barrier and learning curve for inexperienced users, and generally decrease the coding burden allowing users to focus on the biological question and data visualization; (ii) to allow the implementation of modular workflows, giving the possibility to effortlessly try different methodologies and/or algorithms; and (iii) to eliminate the data integration effort necessary for data exploration and visualization, and to enable the direct use of existing powerful tidy visualization tools. Given its simplicity, tidybulk is an effective tool for both scientific and educational purposes.

## Results and discussion

### Data structure

The underlying data structure in tidybulk integrates transcript abundance information and sample-wise or transcript-wise annotation in a tidy format. The only three mandatory columns are sample and transcript identifiers and transcript abundance. Optional columns include biological and technical prior or newly calculated information. In the backend, the information needed for each algorithm is extracted from the tidybulk data frame. The tidybulk data frame is based on the tibble (tbl_df) format [30], which is a modern implementation of the R data frame, offering robustness and ease of visualization. The main advantages of this data structure are (i) consistent subsetting avoids bug-prone behaviors typical of the R native data frame and (ii) the interface with the whole tidyverse ecosystem, including dplyr, tidyr, purrr, magrittr, and a large number of modules being developed by the community. The tidybulk object stores out of sight the key column semantics and raw results for backend algorithms such as PCA, MDS [31], edgeR [13], limma-voom [29], and DESeq2 [16]. The latter can be easily extracted for further custom analyses and diagnostics. A tidybulk data frame (for example, Table 1) can be produced from two sources: (i) a tidy tibble with sample, transcript, abundance, and optional annotation columns and (ii) BAM/SAM files using the function tidybulk_SAM_BAM wrapping featureCounts [12].

**Table 1:** Example of a tidybulk data frame

Sample (fctr)	Transcript (fctr)	Abundance (int)	Annotation	...
S1	CD3G	0	Treated	...
S2	CD3G	100	Treated	...
S3	CD3G	5	Naive	...
S4	CD3G	5240	Naive	...
...	...	...	...	...

### Grammar and API structure

#### Vocabulary

Standard transcriptomic analysis workflows encompass several common procedures, such as scaling of transcript abundance (i.e., normalization), aggregation of isoforms into genes, adjusting for unwanted variation, dimensionality reduction, clustering, cellularity deconvolution, differential abundance analysis, transcript filtering based on variability and/or low relative abundance, identification of redundant samples, and gene enrichment analysis. The tidybulk grammar consistently expresses all steps of the transcriptomics workflow using self-explanatory function names, composed of a specific verb and one or two explanatory terms. All functions can be applied to a tidybulk data frame directly, or any tibble data frame providing the “.sample,” “.transcript,” and “.abundance” arguments as symbolic column names (Table 2).

**Table 2:** Grammar of the functions and packages integrated in the current version 1.1.7 [32]

Name	Description	
**Analysis**		
**Function name**	**Description**	**Integrated packages**
adjust_abundance	Remove known unwanted variation	ComBat [33]
aggregate_duplicates	Summarize the abundance of duplicated transcripts (e.g., isoforms)	
cluster_elements	Identify sample or transcript clusters	Kmeans [34], SNN [20]
deconvolve_cellularity	Identify cell type fraction within each sample	Cibersort [23], EPIC [24], lsfit [35]
identify_abundant	Identify abundant transcripts to be used in subsequent analyses	edgeR [13]
keep_abundant	Filter out rare transcripts	
keep_variable	Filter out non-variable transcripts	limma [31]
reduce_dimensions	Calculate reduced dimensions of transcript abundance	limma [31], PCA [35], Rtsne [21]
remove_redundancy	Filter out redundant samples or transcripts	
scale_abundance	Scale (i.e., normalize) the transcript abundance to compensate for diverse sequencing depth across samples	TMM [14]
test_differential_abundance	Test the hypothesis of differential abundance of transcripts across biological/experimental conditions	edgeR [13], DESeq2 [16], limma-voom [29]
test_gene_enrichment	Test the hypothesis of rank-based enrichment of transcript signatures	EGSEA [36]
test_gene_overrepresentation	Test the hypothesis of gene set enrichment for an unranked gene list	clusterProfiler [26]
test_differential_cellularity	Test the hypothesis of differential tissue composition	lm [35], coxph [17, 37]
**Main utilities**		
get_bibliography	Extract the bibliography for your workflow from any tidybulk object	
impute_missing_abundance	Impute abundance for missing data points using sample groupings	
pivot_sample	Extract non-redundant sample-related information from the data frame	
pivot_transcript	Extract non-redundant transcript-related information from the data frame	
tidybulk	Create a tidybulk data frame from a standard data frame	
tidybulk_SAM_BAM	Infer transcript abundance from mapped reads and create a tidybulk data frame	featureCounts [12]

#### Coding paradigm

The tidybulk functions can either add new sample-wise (e.g., reduced dimensionality) or transcript-wise (e.g., differential transcription statistics) information to the tidybulk data frame in new columns, subset the data frame, or return a standard data frame in case that the function outputs information that is not sample- nor transcript-wise (e.g., enriched gene signatures). Any function that can return a tidybulk data frame can be used in three modes (setting the “action” argument). The mode “add” returns a tidybulk data frame with additional information joined to the input tidybulk data frame; the mode “get” returns a standard data frame with non-redundant sample- or transcript-wise information with the newly calculated information added as new columns, and the mode “only” returns a standard data frame with only the newly calculated information. The “add” mode is used to pass the information across the tidybulk functions, while the mode “get” and “only” are used for independent analysis, manipulation, or visualization outside the tidybulk stream, interfacing with the tidyverse ecosystem. A tidybulk data frame can also be manipulated with tidyverse functions (e.g., dplyr and tidyr) retaining its attributes. To maintain the flexibility that the backend algorithms offer while maintaining the robustness and coding efficiency of the function abstractions, each tidybulk function accepts ellipsis (i.e., … argument in R language) that will be passed as additional arguments to the backend function. For several operations such as dimensionality reduction and differential transcript abundance analysis, the raw output of the underlying algorithms is stored in the data frame attributes.

### Implementing workflows

#### Differential gene transcriptional abundance analysis

**Fig. 1:**
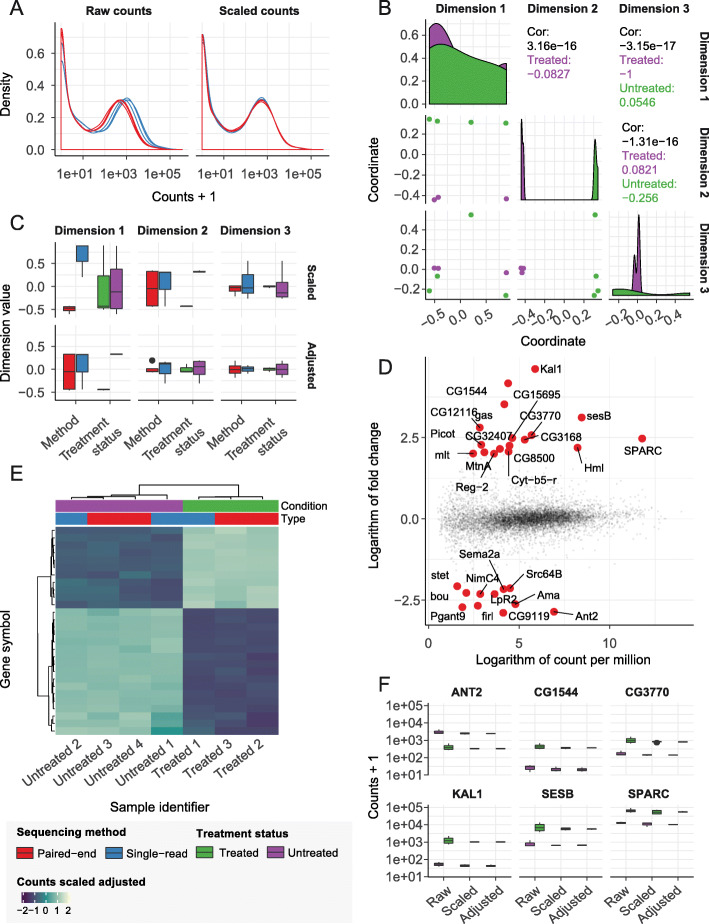
Results for the differential transcript abundance workflow, at the gene level. **a** Density plots of transcript abundance. **b** Pair plot of the first three reduced dimensions. **c** Boxplots of the first three reduced dimensions, before and after removal of unwanted variation. **d** Scatter plot of count per million against fold change. Genes highly differentially transcribed are highlighted in red. **e** Heatmap of the gene transcriptional abundance for the differentially abundant transcripts, at the gene level. **f** Boxplot of the transcript abundance for the top six differentially abundant transcripts at the gene level, for raw, scaled, and adjusted abundance

An important phase of an analysis workflow is data exploration, which involves visualization and production of summary statistics, in combination with dimensionality reduction, data scaling, and adjustment. The following code example illustrates how to produce a tidybulk data frame and how to perform scaling. The transcripts that are duplicated are aggregated and the transcript abundance scaled for sequencing depth is added to the data frame. The default scaling method is edgeR’s TMM [14] but other options are available. The resulting data frame is used to plot the transcript abundance densities for raw or scaled abundance (Fig. 1a) using common tidyverse tools.

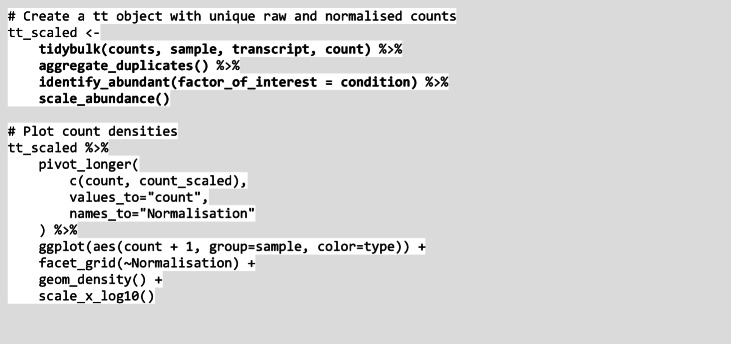



Sample-wise dimensionality reduction using the multidimensional scaling, principal component analysis, or t-distributed stochastic neighbor embedding (MDS, PCA, or tSNE) algorithm [20, 31, 38] can then be performed, as illustrated in the following code example. The information content of sample-wise data can be visualized in three dimensions [39], comparing them against each other (Fig. 1b) using common tidyverse tools.

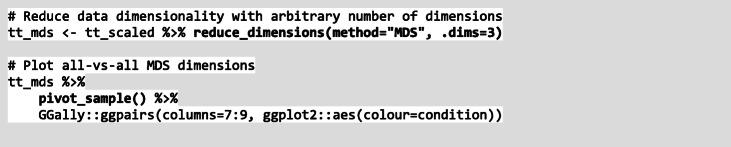



The following code example illustrates how to adjust transcript abundance for known unwanted variation (sequencing type). A formula can be used to define the wanted (first covariate) and unwanted (second covariate) variation [40], using Combat [33]. The reduced dimensions can be calculated again for the adjusted counts, for comparative purposes.

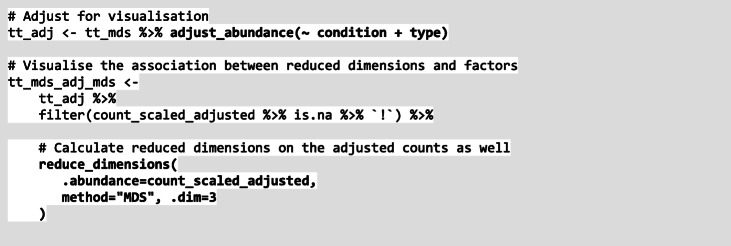



Using tidyverse tools, the tidybulk data frame can be reshaped to create informative comparative plots with little coding burden. The association between biological variability and reduced dimensions can be compared before and after adjustment (Fig. 1c). The plot shows as the first reduced dimension was associated with sequencing technique (technical variability) before adjustment, and with biological category after adjustment.

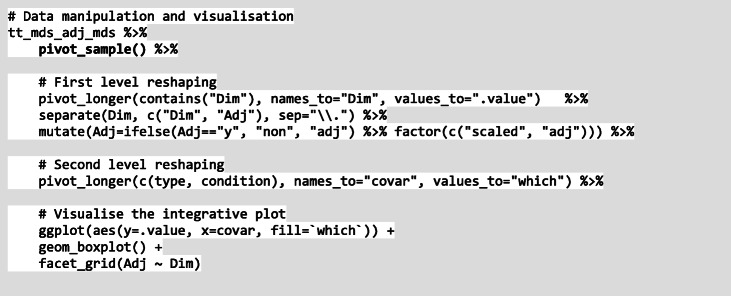



The following code illustrates how to test the association of transcript abundance with the factor of interest. The test can be performed using either edgeR [13], limma-voom [29], or DESeq2 [16]. The relationship between estimated fold change and mean transcript abundance can be visualized with a customized MD plot [31] (Fig. 1d), using common tidyverse tools.

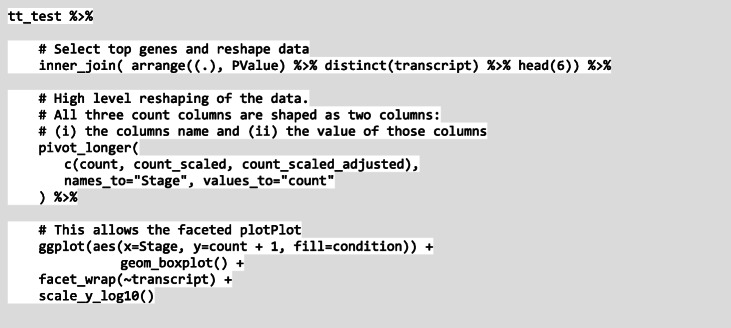



The following code illustrates how to visualize the result of the hypothesis testing. Using tidyverse tools, the resulting tidybulk data frame can be reshaped to visualize the gene transcriptional abundance for the top gene with differential transcript abundance, across several steps of the workflow (raw, scaled, and adjusted abundance; Fig. 1f).

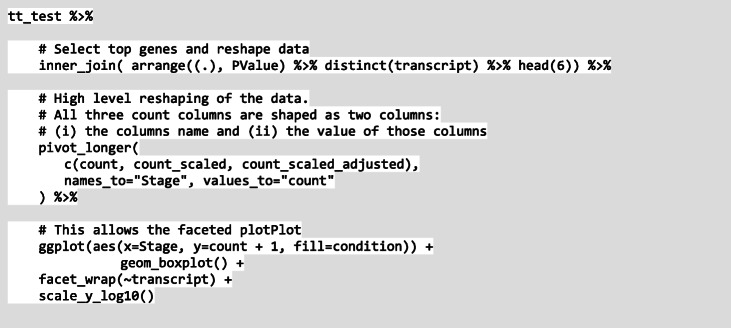



Using the tidyHeatmap package [41], the abundance of the transcripts that are most associated with the factor of interest can be visualized (Fig. 1e).

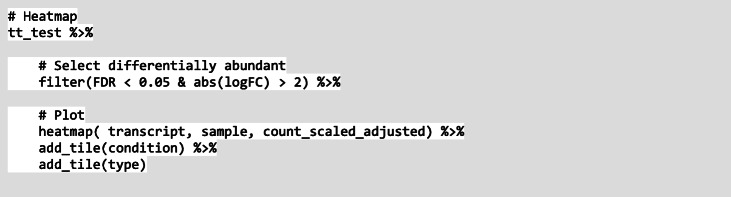



### Identification of transcriptional signature

This workflow showcases the integration of tidybulk and tidyverse tools to select cell-type-specific marker transcripts. This example workflow is aimed at more experienced users, as it shows some advanced integration between tidybulk and tidyverse. The following code example illustrates how to produce a tidybulk data frame from a tibble data frame including transcript abundance of 148 samples representing 16 cell types, collected from public repositories including BLUEPRINT [42], ENCODE [43], GSE77808 [44], PRJNA339309 [45], GSE122325 [46], GSE125887 [47], GSE130379 [48], GSE133478 [49], GSE130286 [50], GSE89442 [51], and GSE107011 [52]. Duplicated transcripts are aggregated, and the abundance of all samples is scaled to compensate for sequencing depth. As the source data comes from diverse sources, the integrated dataset is not rectangular (i.e., same number of sample-transcript pairs). Therefore, we impute the missing data within each cell type category.

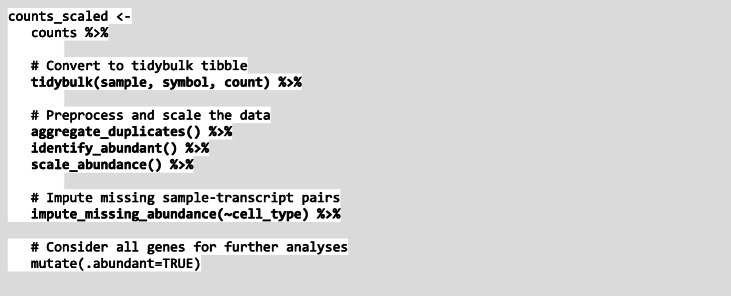



The following code illustrates how to remove redundant samples based on correlation of the top thousand variable transcripts. The rationale is twofolds. First, public repositories often include duplicated samples with different identifiers. Second, we want to avoid that any large study that includes several samples with low biological variability dominates the selection of marker transcripts.

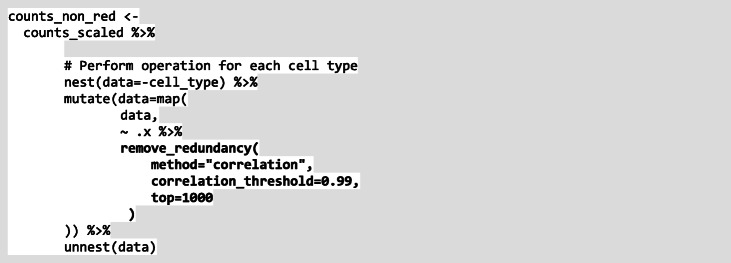



The following code example illustrates how to manipulate the tidybulk data frame with tidyverse tools, to exclude transcripts that are not in all cell types for at least one sample.

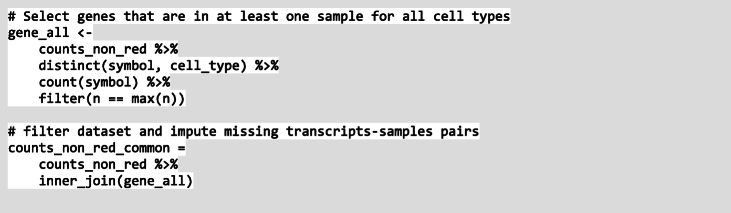



**Fig. 2:**
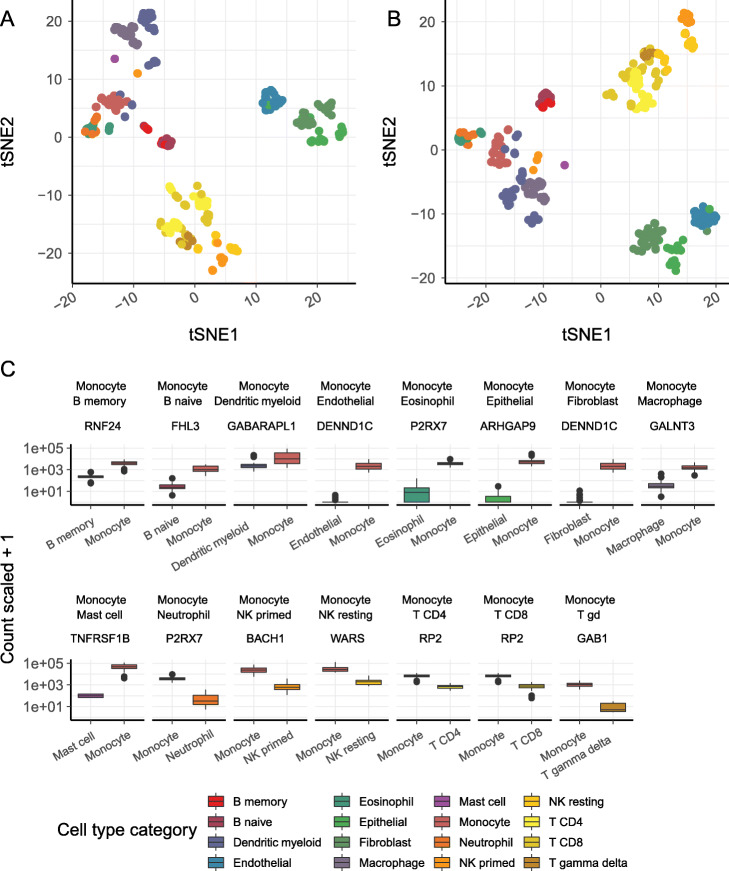
Results for the signature workflow. **a** Scatter plot of the tSNE reduced dimensions for all samples using the most variable transcripts. **b** Scatter plot of the tSNE reduced dimensions for all samples using the variable marker transcripts. **c** Boxplot of the first top marker gene for each comparison between monocytes and all other cell types

The following code illustrates how to visualize the processed data in a reduced dimensional space (Fig. 2a), preserving local similarities using t-distributed stochastic neighbor embedding [53]. The use of tSNE facilitates the visualization of many heterogeneous classes and their internal similarity. This plot can be obtained with an integration of tidybulk and tidyverse tools.

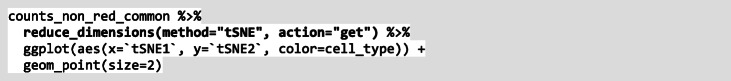



The following code illustrates how to identify transcript markers for each cell type, performing a differential abundance analysis across all the cell type permutations. The strategy used here is to compare all cell types against each other and to select the genes with the largest positive change in transcription, with log fold change larger than two. For each cell-type permutation [54, 55], a set of top markers will be selected. Briefly, a tidy data frame is created for all permutations; then, a function that performs the differential analysis is mapped to each of the permutations. The resulting data frame is filtered for large fold changes and statistical significance.

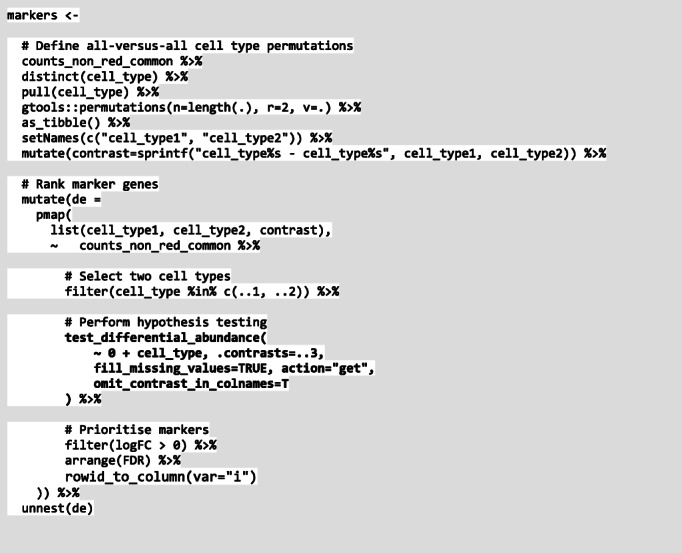



The following code exemplifies how to visualize the difference in transcript abundance of the top monocyte marker against each other cell type.

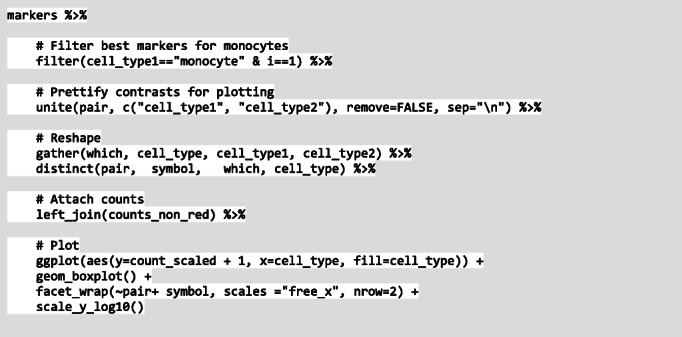



The following code illustrates how to visualize the tSNE reduced dimensions of all samples, using the top marker transcripts. Using those markers, the samples belonging to the same cell type define tighter clusters.

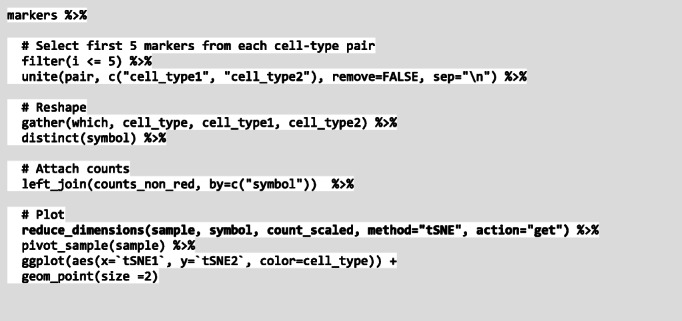



### Coding, memory, and time efficiency

**Fig. 3:**
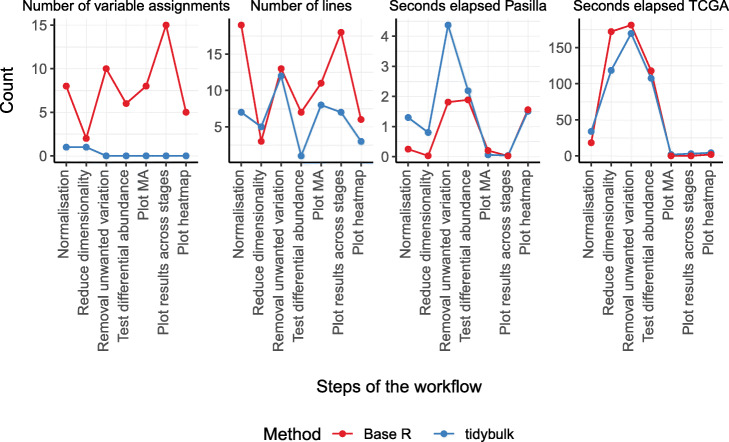
Benchmark for the differential transcript abundance workflow, comparing tidybulk with a standard coding style. Time performances are calculated for the Pasilla [56] and primary prostate TCGA [57] datasets

The workflow for differential transcript abundance analyses at the gene level was used to benchmark tidybulk and base R coding standards on two datasets: Pasilla [56] and primary prostate TCGA [57]. Benchmarking was performed on a Windows machine (12 hyper threads, 32 Gb of RAM) for (i) number of variable assignments, (ii) number of R code lines needed, and (iii) seconds elapsed for each step of the workflow (Fig. 3). Using the tidybulk and tidyverse frameworks, the number of variable assignments needed decreases more than tenfold compared to standard coding style, and the number of lines needed was halved. These two aspects are relevant in interactive programming as both are bug-prone-related factors. Despite the decrease in code complexity and the higher abstraction provided by tidybulk, the time efficiency for the analysis of the Pasilla [56] (small) and primary prostate TCGA [57] (large) datasets is highly comparable with base R coding style (Fig. 3c; using the option tidybulk_do_validate=FALSE).

A redundant tidy data frame has a larger memory footprint compared to multiple disjointed non-redundant data frames. Compared to SummarizedExperiment, a tibble container uses 24% (2.4 Mb compared to 3.1 Mb) more memory for a small annotated datasets such Pasilla [56], 49% (230 Mb compared to 342 Mb) for an unannotated large datasets such as primary prostate TCGA [57], and 4.4 times more (230 Mb compared to 1.4 Gb) for the annotated alternative. Although large-scale datasets can be easily handled on modern personal computers, a future direction is to base tidybulk on a tibble abstraction of the SummarizedExperiment object, rather than a tibble data frame itself. This will enable improvement of the memory footprint of tidybulk without compromising its usability and clarity.

## Conclusions

The analysis of bulk tissue transcriptomic data is grounded in a mature computational ecosystem. However, the research community has not converged on a standard representation of the data nor a user-friendly vocabulary. This represents a limitation for workflow modularity and a high entry barrier for new users. Here with tidybulk, we introduce a tidy representation of transcriptomics data that explicitly conveys the relation between biological, clinical, and statistical quantities, and can harvest the data manipulation capabilities of the tidyverse ecosystem. Furthermore, the endomorphic properties (i.e., that do not change in the input-output stream) of this data structure enable modularity of the workflow steps. This makes it easy to add or drop analysis steps and to test alternative analysis algorithms for each step of the workflow. Our framework manipulates and analyzes this data using robust wrapper functions for a wide variety of processes that are common in transcriptome analyses. Similarly to tidyverse, these wrappers use a clear and self-explanatory grammar. The bridge between tidy data representation and compatibility with the tidyverse allows publication-ready data visualization. Although our framework was developed for end-users, we aim to create an integration and validation API allowing developers to expand the framework with more functionality. Due to its simplicity and intuitive grammar, we anticipate that tidybulk will also be suitable for educational purposes.

## Supplementary Information

**Additional file 1.** Review history.

## Data Availability

Tidybulk is available on GitHub github.com/stemangiola/tidybulk, and on Bioconductor bioconductor.org/packages/release/bioc/html/tidybulk.html. The version used for this article is 1.1.7 [32]. The code is released under the version 3 of the GNU General Public License. The web page of the tidybulk package is stemangiola.github.io/tidybulk. The example code included in this manuscript is available as a markdown file at github.com/stemangiola/tidybulk/vignettes. The code used for benchmarking is available at github.com/stemangiola/tidybulk in the directory dev/benchmark. The datasets used to benchmark the differential gene transcriptional abundance workflow are Pasilla [56] and the primary prostate dataset from The Cancer Genome Atlas (TCGA) [57]. The datasets used for transcriptomic signature workflow were BLUEPRINT [42], ENCODE [43], GSE77808 [44], PRJNA339309 [45], GSE122325 [46], GSE125887 [47], GSE130379 [48], GSE133478 [49], GSE130286 [45], GSE89442 [46], and GSE107011 [47].
