# Supplementary material for: tidybulk: an R tidy framework for modular transcriptomic data analysis
Source: Genome Biol. 2021 Jan 22;22:42. doi: 10.1186/s13059-020-02233-7 (PMC7821481; doi:10.1186/s13059-020-02233-7)
Supplement: Supplementary file 1 — Additional file 1. Review history. [file 13059_2020_2233_MOESM1_ESM.docx]

Review History

**First round of review**

**Reviewer 1**

**Were you able to assess all statistics in the manuscript, including the appropriateness of statistical tests used?**

Yes, and I have assessed the statistics in my report.

**Were you able to directly test the software?**

Yes

**Comments to author:**

The authors present a new framework and software package within Bioconductor for tidyverse-style manipulation of transcriptomics data. The authors provide a number of examples demonstrating how various complex analyses can be performed using dplyr-like manipulations of objects, thus avoiding many temporary variables. The approach described is novel, as the existing publication in this journal that brings tidyverse-style syntax to Bioconductor objects, plyranges, is designed for manipulation of genomic range objects, while tidybulk focuses on manipulation of gene expression data (typically stored in gene-by-sample matrices). I was able to download/install and to run the tidybulk functions on my computer.

The approach described is likely of value to the community of bioinformatic analysts, both for workflows and for tutorials/teaching, although I have a few reservations described below.

Major Comments:

1) The current functions to some degree obfuscate the underlying function calls and the normalization and statistical testing methods being employed. While it is clear from documentation that `scale_abundance` will make use of edgeR::calcNormFactors, it was not entirely clear from the documentation what goes on between `adjust_abundance` and `test_differential_abundance`. How does the user know that `test_differential_abundance` will not use the adjusted values from `adjust_abundance`? For example, this was a bit confusing from the existing code because it appears that the sequencing type variation was removed in the adjustment step via ComBat, but then also included in the testing step as a nuisance covariate. This is just an example, but in general the wrapper functions may obfuscate to some degree the underlying function calls, which the user may need to access in order to understand what exactly is happening to the data, and to report them in manuscripts. One possible solution may be if the underlying edgeR function calls were somehow communicated to the user or stored in a systematic way.

2) The authors mention that the software allows for modularity, but the fact that the functions wrap a limited set of commands specific to edgeR, a particular Bioconductor package, mean that it is not clear how alternative analysis workflows would be possible. For example, could one run a limma-voom or DESeq2 analysis? Could one access the alternatives within edgeR, robust dispersion estimation or the quasi-likelihood? For gene set testing, it appears that only the ensemble method is supported, so one cannot use specifically, e.g. roast or camera.

3) It is mentioned that the tidy data frame requires more memory. How much more, compared to standard, on datasets of various size?

4) In Figure 3, the timing is presented for various tasks. For some of these, the timing seems to be substantially longer. How does tidybulk scale then? On the prostate cancer TCGA data set mentioned at the end of the article (600 Mb), what is the comparison in terms of time to perform the first three operations in Figure 3?

Minor comments:

Some of the code chunks are not very easy to follow, even for someone with experience with edgeR and tidyverse commands, some examples are the `pivot_longer` command in code chunk 6, and the `map_int` command in code chunk 12. I would recommend to simplify the examples to further support the idea that the tidy style is more clear and self-explanatory for these analyses.

Code should be made available for Figure 3's comparisons.

I wasn't able to access the vignette on the Bioconductor website at the time of this review (May 12).

**Reviewer 2**

**Were you able to assess all statistics in the manuscript, including the appropriateness of statistical tests used?**

There are no statistics in the manuscript.

**Were you able to directly test the software?**

Yes

**Comments to author:**

Overall, the paper is nicely written and makes a significant contribution to the field.

-Is the Software likely to be of broad utility? Is it easy to install and use?

The software was easy to install from the Bioconductor site. The example code provided with the package works without modification, although throws many warnings. The code embedded in the paper cannot be easily extracted and checked that it runs. It's not clear that the figures can be reproduced from the code from the paper, because of this.

-Does the software represent a significant advance over previously published tools (as demonstrated by direct comparison with available related software)?

This software represents another step towards streamlining bioinformatics data analysis, that will make it easier for a broader base of bioinformaticians to utilise. My main concerns are that the previous work in this area has not been sufficiently referenced. I have listed these below. In particular, a comparison of this work needs to be made with the methods available in the plyranges package.

-Please indicate briefly the novel features and/or advantages of the software and/or please reference the relevant publications and which alternatives, if any, it should be compared with.

The tidy approach to working with data provides an efficiency by reducing the human cognitive load. Instead of the analyst having to conform to the software syntax, and grapple with many different standards from different software developers, the software interface is standardized. The language also better maps to the data processing steps, making it easier to follow the flow of the analysis, and the steps taken. This assists in reproducbility, because it is easier for others to digest what the analysts have done. This work is a new contribution in this area, and represents an emerging area of research.

-Is the paper of broad interest to others in the field, or of outstanding interest to a broad audience of biologists?

This work should be of broad interest to biologists because it will make it easier to do sophisticated data analysis with fewer computer science skills. Biologists will be able to better explain and rationalise their analyses with tools like this.

This a list of work that needs to be citied:

Lee, Stuart, Cook, Dianne, Lawrence, Michael (2019). "plyranges: a grammar of genomic data transformation." Genome Biol., 20(1), 4. http://dx.doi.org/10.1186/s13059-018-1597-8.

Gregory R. Warnes, Ben Bolker and Thomas Lumley (2020). gtools: Various R Programming Tools. R package version 3.8.2. https://CRAN.R-project.org/package=gtools (Permutation testing is conducted using this package, but the package is not cited. Any other R packages that are used in your work should also be cited.)

Bůžková, P., Lumley, T., & Rice, K. (2011). Permutation and parametric bootstrap tests for gene-gene and gene-environment interactions. Annals of human genetics, 75(1), 36-45. https://doi.org/10.1111/j.1469-1809.2010.00572.x

Yin T, Cook D, Lawrence M (2012). "ggbio: an R package for extending the grammar of graphics for genomic data." Genome Biology, 13(8), R77. (This discusses a grammar for biological data plots, which should be discussed in relation to the plots in this paper.)

Kirill Müller and Hadley Wickham (2020). tibble: Simple Data Frames. R package version 3.0.2. https://CRAN.R-project.org/package=tibble (This is the appropriate citation for the tibble work, not the one that you have used in your paper. Actually, the paper that you have cited is a really bad mistake, because this is someone who is simply telling other people that they need to use tibble - its a secondary source and that author made no contribution to the tibble concepts. You should make sure that you have not made similar mistakes with other citations.)

**Authors Response**

**Point-by-point responses to the reviewers’ comments:**

Response to Reviewers

*We thank the reviewers for their careful and constructive feedback. We have carefully addressed each of the reviewers’ points below.*

*We note that we have added extensive additional functionality to tidybulk including support for DeSeq2, both the quasi-likelihood and likelihood ratio methods from edgeR, and LIMMA-VOOM for testing of differential transcript abundance; COMBAT for removal of unwanted variation; CIBERSORT, lsfit and EPIC for estimating tissue composition; as well as gene set enrichment testing using multiple methods from EGSEA, and ROAST and CAMERA from LIMMA; and clusterProfiler for functional profiling. So tidybulk now supports the 3 major R toolkits for differential transcript expression analysis using a single interface, as well as many additional tools for handling transcriptomic data. The development of tidybulk continues at a rapid pace.*

*We have added extensive additional citations of relevant methods into the manuscript, including plyranges and other packages as requested by the reviewers. We have also extended the documentation to clarify what tools are being used.*

*We hope that you find the tidybulk manuscript suitable for publication.*

Reviewer #1:

The authors present a new framework and software package within Bioconductor for tidyverse-style manipulation of transcriptomics data. The authors provide a number of examples demonstrating how various complex analyses can be performed using dplyr-like manipulations of objects, thus avoiding many temporary variables. The approach described is novel, as the existing publication in this journal that brings tidyverse-style syntax to Bioconductor objects, plyranges, is designed for manipulation of genomic range objects, while tidybulk focuses on manipulation of gene expression data (typically stored in gene-by-sample matrices). I was able to download/install and to run the tidybulk functions on my computer.

The approach described is likely of value to the community of bioinformatic analysts, both for workflows and for tutorials/teaching, although I have a few reservations described below.

Major Comments:

1) The current functions to some degree obfuscate the underlying function calls and the normalization and statistical testing methods being employed. While it is clear from documentation that `scale_abundance` will make use of edgeR::calcNormFactors, it was not entirely clear from the documentation what goes on between `adjust_abundance` and `test_differential_abundance`. How does the user know that `test_differential_abundance` will not use the adjusted values from `adjust_abundance`? For example, this was a bit confusing from the existing code because it appears that the sequencing type variation was removed in the adjustment step via ComBat, but then also included in the testing step as a nuisance covariate. This is just an example, but in general the wrapper functions may obfuscate to some degree the underlying function calls, which the user may need to access in order to understand what exactly is happening to the data, and to report them in manuscripts. One possible solution may be if the underlying edgeR function calls were somehow communicated to the user or stored in a systematic way.

*Thank you for this feedback, which we totally agree with. We have tried to address this though multiple improvements detailed below.*

*In order to gain transparency about the procedures in the back-end, the documentation of all tidybulk methods now clearly describes which algorithms the methods are based on, and displays the key R code chunks to give to the user the sense of the information flow and how the algorithms are called.*

*So that all methods used are appropriately cited in manuscripts of users, every tidybulk object now tracks all algorithms used for its creation, manipulation and analysis (obj %>% attr("internals") %$% methods_used), and a new method has been added to print the whole bibliography (obj %>% get_bibliography()). This not only adds clarity but also allows the user to easily acknowledge the contribution of the scientific community.*

*In order to increase transparency and flexibility for expert users, tidybulk stores the raw objects produces by backend algorithms (e.g. PCA object), and a message is clearly displayed for each step of the workflow about how to extract such an object (e.g. obj %>% attr("internals") %$% PCA). This allows the users to further analyse the diagnostics of results that are not displayed within the output data frame.*

*The clear separation of normalisation/adjustment from hypothesis testing (test_differential_abundance) is a major concern for us, as the latter is a key step of transcriptomic analysis workflows. We are well aware that this point has to be made extremely clear in the documentation. We added the sentence “All methods use raw counts, irrespective of whether scale_abundance or adjust_abundance have been calculated, therefore it is essential to add covariates such as batch effects (if applicable) in the formula.” both in the help pages as well as a message printed to the user each time the test_differential_abundance method is called.*

2) The authors mention that the software allows for modularity, but the fact that the functions wrap a limited set of commands specific to edgeR, a particular Bioconductor package, mean that it is not clear how alternative analysis workflows would be possible. For example, could one run a limma-voom or DESeq2 analysis? Could one access the alternatives within edgeR, robust dispersion estimation or the quasi-likelihood? For gene set testing, it appears that only the ensemble method is supported, so one cannot use specifically, e.g. roast or camera.

*We agree. Since the first submission of the manuscript we have added multiple additional packages. It is our goal to include the primary/most requested software in the tidubulk framework.*

*The function test_differential_abundance now includes edgeR quasi likelihood, edgeR likelihood ratio, DESeq2 and Limma-voom as pipelines. With adding an optional prefix, we made it easy to run all methods of choice and quickly compare the results visually or analytically.*

*For gene enrichment testing, the user can choose one or more methods of choice within the underlying EGSEA, such as Roast and Camera. Moreover, the algorithm clusterProfiler is now also available under test_gene_overrepresentation. Several algorithms are now included in the cellularity deconvolution API, including Cibersort, lsfit and EPIC, and both standard and cox regressions are applicable as hypothesis testing using the function test_differential_cellularity.*

*As a note, in the future we plan to include an API for developers. This API will allow developers and users to add arbitrary methods to the framework, offering input/output validation. This high-level API however requires thorough discussion with the community and software-engineer experts, and a higher maturity of the framework. It is however a clear direction of ours.*

3) It is mentioned that the tidy data frame requires more memory. How much more, compared to standard, on datasets of various size?

*The increase in memory is dataset dependent. In order to estimate the increase on a typical dataset, we added two examples of SummarizedExperiment and tibble object sizes for a small (pasilla) and a big (PRAD TCGA) datasets.*

*We added the following text “A redundant tidy data frame generally has a larger memory footprint compared to multiple disjointed non-redundant data frames. Compared to SummarizedExperiment, a tibble container uses 24% (2.4Mb compared to 3.1Mb) more memory for a small annotated datasets such Pasilla (Brooks et al. 2011); 49% (230Mb compared to 342Mb) for unannotated large datasets such as PRAD TCGA (The Cancer Genome Atlas Program 2018); and 4.4 times more (230Mb compared to 1.2Gb) for the annotated (5 extra columns) PRAD TCGA dataset. Although large scale datasets can be easily handled on modern personal computers, a future direction is to base tidybulk on a tibble abstraction of the SummarizedExperiment object, rather than a tibble data frame itself. This will allow to improve the memory footprint of tidybulk without compromising its usability and clarity.” was added to the “Coding, memory and time efficiency” sub-section.*

*As a note and as mentioned in the manuscript, our future goal is to base tidybulk on a tibble abstraction of the SummarizedExperiment object, rather than the tibble data frame itself. In the near future we expect the memory footprint of tidybulk will match the one of SummarizedExperiment.*

4) In Figure 3, the timing is presented for various tasks. For some of these, the timing seems to be substantially longer. How does tidybulk scale then? On the prostate cancer TCGA data set mentioned at the end of the article (600 Mb), what is the comparison in terms of time to perform the first three operations in Figure 3?

*We thank Reviewer #1 for this comment. Figure 3 has been updated with the benchmark for the primary prostate TCGA datasets. As the size of the dataset increases the gap between base R and tidybulk decreases.*

Minor comments:

Some of the code chunks are not very easy to follow, even for someone with experience with edgeR and tidyverse commands, some examples are the `pivot_longer` command in code chunk 6, and the `map_int` command in code chunk 12. I would recommend to simplify the examples to further support the idea that the tidy style is more clear and self-explanatory for these analyses.

*The two examples mentioned by Reviewer #1 has been simplified and better commented.*

*Block 6 now reads*

*“[...]*

*# High level reshaping of the data.*

*# All three count columns are shaped as two columns:*

*# (i) the columns name and (ii) the value of those columns*

*pivot_longer(*

*c(count, count_scaled, count_scaled_adjusted),*

*names_to = "Stage", values_to = "count"*

*) %>%*

*[...]”*

*From block 12 map_int has been dropped, and now reads*

*“markers <-*

*# Define all-versus-all cell type permutations*

*counts_non_red_common %>%*

*distinct(cell_type) %>%*

*pull(cell_type) %>%*

*gtools::permutations(n = length(.), r = 2, v = .) %>%*

*as_tibble() %>%*

*setNames(c("cell_type1", "cell_type2")) %>%*

*mutate(contrast = sprintf("cell_type%s - cell_type%s", cell_type1, cell_type2)) %>%*

*# Rank marker genes*

*mutate(de =*

*pmap(*

*list(cell_type1, cell_type2, contrast),*

*~	counts_non_red_common %>%*

*# Select two cell types*

*filter(cell_type %in% c(..1, ..2)) %>%*

*# Perform hypothesis testing*

*test_differential_abundance(*

*~ 0 + cell_type, .contrasts = ..3,*

*fill_missing_values = TRUE, action="get",*

*omit_contrast_in_colnames = T*

*) %>%*

*# Prioritise markers*

*filter(logFC > 0) %>%*

*arrange(FDR) %>%*

*rowid_to_column(var = "i")*

*)) %>%*

*unnest(de)”*

*The examples are generally better explained and simplified when possible.*

*As a note, we have attempted to provide both simple and advanced examples in the manuscript for different users. We would prefer to retain this diversity and to offer something for both less and more experienced users. We have noted this in the text leading up to the example code in the subsection “Data exploration and differential transcript abundance”.*

Code should be made available for Figure 3's comparisons.

*The code is now available in the code repository https://github.com/stemangiola/tidybulk in the directory dev/benchmark. This is now explicit in the subsection “Code availability”.*

I wasn't able to access the vignette on the Bioconductor website at the time of this review (May 12).

*The vignette is now accessible.*

Reviewer #2:

Overall, the paper is nicely written and makes a significant contribution to the field.

-Is the Software likely to be of broad utility? Is it easy to install and use?

The software was easy to install from the Bioconductor site. The example code provided with the package works without modification, although throws many warnings. The code embedded in the paper cannot be easily extracted and checked that it runs. It's not clear that the figures can be reproduced from the code from the paper, because of this.

*We apologise for this inconvenience. The warnings have been eliminated and the code for the chunks is available in the vignette https://github.com/stemangiola/tidybulk/vignettes/manuscript* for copying and pasting.*

-Does the software represent a significant advance over previously published tools (as demonstrated by direct comparison with available related software)?

This software represents another step towards streamlining bioinformatics data analysis, that will make it easier for a broader base of bioinformaticians to utilise. My main concerns are that the previous work in this area has not been sufficiently referenced. I have listed these below. In particular, a comparison of this work needs to be made with the methods available in the plyranges package.

This a list of work that needs to be cited:

Lee, Stuart, Cook, Dianne, Lawrence, Michael (2019). "plyranges: a grammar of genomic data transformation." Genome Biol., 20(1), 4. http://dx.doi.org/10.1186/s13059-018-1597-8.

Gregory R. Warnes, Ben Bolker and Thomas Lumley (2020). gtools: Various R Programming Tools. R package version 3.8.2. https://CRAN.R-project.org/package=gtools (Permutation testing is conducted using this package, but the package is not cited. Any other R packages that are used in your work should also be cited.)

Bůžková, P., Lumley, T., & Rice, K. (2011). Permutation and parametric bootstrap tests for gene-gene and gene-environment interactions. Annals of human genetics, 75(1), 36-45. https://doi.org/10.1111/j.1469-1809.2010.00572.x

Yin T, Cook D, Lawrence M (2012). "ggbio: an R package for extending the grammar of graphics for genomic data." Genome Biology, 13(8), R77. (This discusses a grammar for biological data plots, which should be discussed in relation to the plots in this paper.)

Kirill Müller and Hadley Wickham (2020). tibble: Simple Data Frames. R package version 3.0.2. https://CRAN.R-project.org/package=tibble (This is the appropriate citation for the tibble work, not the one that you have used in your paper. Actually, the paper that you have cited is a really bad mistake, because this is someone who is simply telling other people that they need to use tibble - its a secondary source and that author made no contribution to the tibble concepts. You should make sure that you have not made similar mistakes with other citations.)

*We apologise for this oversight. We have now cited the suggested articles. We have also provided details on additional packages now supported and cite all methods incorporated into the current version of tidybulk.*

**Second round of review**

**Reviewer 1**

The authors have addressed all of my previous concerns.

In particular, they have added underlying code to the documentation of the functions so the user can easily determine what "base Bioconductor" code was used for each tidybulk command for debugging/reporting/reproducibility. One small note (which does not merit additional review) is that the rendering of the code in the documentation is not 1-1 with the code, e.g. the spacing and the pipe commands are being lost. But anyway the authors have taken great effort to provide this information in the man pages.

Secondly, there are a number of impressive additions to the package, include 1) support for limma-voom, edgeR QL and DESeq2, 2) the tracking of algorithms used via the "internals" attribute and ability to print a bibliography, and 3) the storage of the objects produces by backend algorithms. These are great innovations for the tidybulk workflow.

The authors have also added sufficient details regarding the additional memory footprint of the tidybulk objects to the revised manuscript, and the advanced examples are now easier to follow than previously and have inline comments.
